# An integrated roadmap of European sea bass (*Dicentrarchus labrax*) spermatogenesis across the annual reproductive cycle

**DOI:** 10.3389/fcell.2026.1852477

**Published:** 2026-06-24

**Authors:** Nerea Sanchis, Noelia Díaz, Mercedes Blázquez

**Affiliations:** Institute of Marine Sciences (ICM-CSIC), Barcelona, Spain

**Keywords:** chromatin remodelling, European sea bass, flagellum assembly, germ cell differentiation, spermatogenesis, testis biology, transcriptomics

## Abstract

European sea bass (*Dicentrarchus labrax*) is a seasonal marine teleost of high aquaculture value, yet a comprehensive, stage-resolved molecular description of its spermatogenic cycle has been lacking. Here, we generated an integrated histology-anchored RNA sequencing atlas covering the six canonical testicular stages (Stages I-VI) to reconstruct the temporal sequence of endocrine, proliferative, meiotic, spermiogenic and regression-related processes across the annual reproductive cycle. Early stages (Stages I-III) were characterised by strong activation of spermatogonial maintenance pathways, hormone-responsive programmes, and circadian regulators, along with robust enrichment of DNA replication, kinetochore assembly, and chromosomal segregation mechanisms that prepare germ cells for meiotic entry. Spermiogenesis (Stage IV) represented the major transcriptional shift, with the coordinated upregulation of axonemal motors, intraflagellar transport and BBSome components, outer dense fibres and microtubule regulators, accompanied by extensive chromatin remodelling involving histone variants, transcription factors, ubiquitin/SUMO pathway enzymes, and heterochromatin-associated proteins. Spermiation (Stage V) showed an enrichment in calcium-dependent signalling, cytokine and chemokine networks, and adhesion and extracellular matrix pathways, consistent with Sertoli-germ cell remodelling and sperm release. Regression (Stage VI) was dominated by immune activation, endocytosis, phagocytosis, and vascular remodelling signatures, indicating an active phase of clearance and tissue reorganisation that preserves the spermatogonial reservoir for the next cycle. From 9,327 differentially expressed genes, we identified 132 distinct temporal expression patterns and curated three translational resources: stage-informative markers, a fertility gene set, and a chromatin-associated compendium. Together, this atlas defines the sequential molecular architecture of spermatogenesis in a seasonal marine fish, conserved vertebrate and species-specific regulatory modules, and provides operational biomarkers for broodstock assessment and the mitigation of precocious maturation in aquaculture.

## 1 Introduction

Aquaculture is the fastest-growing animal food production sector worldwide and plays a central role in meeting the rising demands for high-quality protein as wild fish stocks continue to decline (FAO, 2025). In Mediterranean aquaculture, the European sea bass (*Dicentrarchus labrax*) is a marine species of major economic relevance, but it exhibits persistent reproductive challenges under farming conditions. These include strongly male-biased sex ratios (75%–95%; Piferrer et al., 2005) and a high incidence of precocious maturation during the first year of life (20%–30%; Felip et al., 2001; Zanuy et al., 2001; Begtashi et al., 2004; Felip et al., 2006). Such traits reduce growth potential and size homogeneity, making the optimisation of reproductive control a key priority in aquaculture.

Teleost spermatogenesis is a highly ordered process driven by coordinated interactions between germ cells and somatic lineages, progressing through spermatogonial proliferation, meiosis, and spermiogenesis to ultimately produce mature spermatozoa (Schulz et al., 2010; Schulz et al., 2023). European sea bass testes exhibit the cystic organisation typical of teleosts, with synchronised germ-cell cohorts enclosed within cysts (Uribe et al., 2014) and six histologically defined stages spanning immature testis, recrudescence, spermiation, and post-spawning regression (Begtashi et al., 2004). Although several molecular studies have addressed aspects of early gonadal development (Viñas and Piferrer, 2008; Crespo et al., 2013) and environmental or endocrine factors influencing reproductive physiology, including temperature-dependent sex differentiation and precocious puberty (Díaz and Piferrer, 2015; Valero et al., 2015; Blázquez et al., 2017; Medina et al., 2019; Barrachina et al., 2018; Ribas et al., 2019), a comprehensive, stage-resolved transcriptomic framework covering the full annual spermatogenic cycle has been lacking.

High-throughput transcriptomics has revealed remarkable cellular and molecular complexity in teleost testes. In asynchronous species such as zebrafish (*Danio rerio*) and medaka (*Oryzias latipes*), single-cell and bulk RNA sequencing (RNA-seq) studies have identified cell-type markers, niche regulators, and signalling networks underlying germ-cell progression (Adolfi et al., 2016; Adolfi et al., 2021; Qian et al., 2022; Burgos-Ruíz et al., 2025). Equivalent datasets in seasonal breeders or marine species, including black rockfish (*Sebastes melanops*; Jin et al., 2024), Korean rockfish (*Sebastes schlegelii*; Wang X. et al., 2023), Chinese tongue sole (*Cynoglossus semilaevis*; Wang H-Y. et al., 2023), large yellow croaker (*Larimichthys crocea*: Yang et al., 2024), and gilthead seabream (*Sparus aurata*; Castro-Arnau et al., 2022), have uncovered conserved pathways related to steroidogenesis, cell-cycle control, spermiogenesis and somatic-germ cell communication (Zhang et al., 2016; Roy et al., 2017; Luo et al., 2019; Castro-Arnau et al., 2022; Wang X. et al., 2023; Jin et al., 2024). However, despite these advances, no study has integrated histological staging with transcriptomic profiling across the entire annual reproductive cycle of European sea bass.

Such an atlas is essential for understanding how endocrine cues, local paracrine factors, circadian components, chromatin-remodelling programmes, cytoskeletal transitions and immune-mediated tissue remodelling are temporally coordinated in a seasonal teleost. It would also provide valuable molecular markers for broodstock assessment and early detection of reproductive activation, with direct applications in mitigating precocious maturation in aquaculture.

Here, we address this gap by integrating detailed histological staging with RNA-seq of testes across the six canonical stages, Stage I (SI) – Stage VI (SVI), of the annual spermatogenic cycle in European sea bass. Through differential expression analysis, temporal clustering, and functional enrichment, we reconstruct the molecular transitions underlying spermatogonial maintenance, meiotic licensing, spermiogenesis, spermiation, and regression. We also identify stage-informative markers and curated gene sets related to fertility and chromatin regulation. This integrated atlas provides a comprehensive molecular framework for spermatogenesis in a seasonal marine fish and offers actionable resources and diagnostic tools for mechanistic reproductive biology and aquaculture management.

## 2 Methods

### 2.1 Experimental animals and husbandry conditions

Sexually mature European sea bass males were maintained under natural photoperiod and temperature conditions (13–25 ± 1 °C following seasonal oscillations), and fed *ad libitum* with commercial pellets (EFICO 4057, BioMar Iberia S.A.) at the Aquarium Facility (ZAE) of the Institute of Marine Sciences (ICM-CSIC), Barcelona (41°23′37″N; 2°10′28″E).

Fish were sampled during their second year of life, corresponding to the period of first sexual maturation in male European sea bass. In this species, the first spermatogenic cycle spans a complete annual sequence from an immature testicular state to spermiation and regression. Importantly, chronological age and gonadal developmental stage are not strictly synchronised in European sea bass, and individuals of the same age cohort may occupy different spermatogenic stages. Consequently, although all sampled males were of similar age, testicular stages assignment was not based on age but on detailed histological criteria, allowing the accurate identification of Stage I (SI) to Stage VI (SVI) testes within the same cohort.

At each sampling point, fish were netted, anesthetised with 2-phenoxyethanol (0.2 mL·L^−1^; Sigma-Aldrich), and euthanised by spinal transection following institutional and national ethical requirements.

### 2.2 Ethics statement

All procedures complied with Spanish regulations (RD53/2013) and the European Directive 2010/63/EU for the protection of animals used for scientific purposes. Experiments were approved by the Ethics Committee of the Local Government of the Generalitat de Catalunya (protocol ref. 10209).

Randomisation was not applicable because sampling was based on predefined reproductive stages. Blinding was not feasible for histological classification, but was applied during RNA extraction and library preparation. No animals were excluded.

### 2.3 Histology and staging of spermatogenesis

Testis fragments were fixed in 4% paraformaldehyde in PBS for 24 h, washed, and dehydrated through a graded ethanol series (70%–96%). Tissues were embedded in Histoplast Paraffin IM (Thermo Scientific MI, United States), sectioned at 7 μm (Leica Reichert-Jung 2040 Autocut Rotary Microtome), and stained with haematoxylin-eosin (Mayer’s hemalum and Eosin Y alcoholic solution 0.5%).

Sections were examined under a Zeiss Axiophot 2 microscope and imaged with an Axiocam MRC 5 camera (Zeiss, Germany). Testicular stages were classified as described by Begtashi et al. (2004). Testicular staging was performed exclusively on the basis of histological criteria, following the canonical descriptions established for European sea bass (Begtashi et al., 2004).

To ensure that the present manuscript is fully self-contained, the criteria used to distinguish germ-cell types and assign spermatogenic stages are summarised here. Each spermatogenic stage is defined by the composition of germ-cell cysts rather than by age. Briefly, six developmental canonical stages were defined: Stage I (SI) corresponds to immature testes containing proliferating type A spermatogonia (SpgA); Stage II (SII; early recrudescence) is characterised by the appearance of type B spermatogonia (SpgB) and some cysts of early primary spermatocytes I (SpcI); Stage III (SIII; mid-recrudescence) by SpgA, SpgB and abundant cysts of primary (SpcI) and secondary (Spc II) spermatocytes indicative of meiotic progression; Stage IV (SIV; spermiogenesis) by the presence of all germ-cell types and active spermiogenesis (including spermatids (Spt) and spermatozoa (Spz)); Stage V (SV; spermiation) by lobules filled with spermatozoa during spermiation; and Stage VI (SVI; regression) by regressed lobules containing residual spermatozoa, an increasing number of SpgA intermingled within the epithelial/connective tissue, and cessation of spermatogenesis. This histology-based classification enabled the identification of SI-SVI testes within a single age cohort. Counts of germ-cell types (SpgA, SpgB) and cysts (SpcI, SpcII, Sptd, and Spz) in nine non-overlapping fields across the six spermatogenic stages are provided in Supplementary Table S1, further supporting the histology-based classification used for all downstream transcriptomic analyses.

### 2.4 RNA extraction, library preparation, and sequencing

Total RNA was extracted from ∼30 mg of frozen tissue using the RNeasy Plus Mini Kit (Qiagen, United States) with on-column DNase digestion (TURBO DNA-free kit, Invitrogen). RNA purity and quantity were assessed with a NanoDrop spectrophotometer (Nanodrop Technologies, United States), and integrity with an Agilent 2100 Bioanalyzer (Agilent Technologies, United States) (RIN 7.0–9.1).

Eighteen samples (six stages with three biological replicates per stage) were used for library preparation. One microgram of total RNA per sample was processed using the QIAseq Stranded RNA Library Kit (QIAGEN, Germany, cat. no. 180450). Library quality was assessed via Bioanalyzer, and sequencing was performed on an Illumina NovaSeq 6000 platform (150 bp paired-end), yielding 40.79–48.98 M paired reads per library (Supplementary Table S2). Reads containing > Ns or >50% bases with Phred <5 were discarded. RNA-seq libraries exhibited high quality across stages (median Q30 > 91%, consistent GC%, low error rate).

### 2.5 *de novo* transcriptome assembly and annotation

Trimmed reads were assembled *de novo* using Trinity v2.6.6 (Inchworm, Chrysalis, Butterfly; default k-mer = 31; Haas et al., 2013). Assembly completeness was quantified using BUSCO v3.0.2 (Manni et al., 2021) against the Actinopterygii dataset.

Transcript annotation integrated multiple databases and pipelines: NCBI NR/NT, Pfam, GOG/eggNOG, Swiss-Prot, Kyoto Encyclopedia of Genes and Genomes (KEGG), Gene Ontology (GO), and the European sea bass reference genome (GCF_905237075.1). Unannotated transcripts were further analysed using UniProtKB/Swiss-Prot, InterPro, Pfam, and BiomaRt (Durinck et al., 2009).

### 2.6 Quantification and differential expression

Reads were clustered with Corset (Davidson and Oshlack, 2014), and quantified using RSEM v1.2.28 (Li and Dewey, 2011) with parameters: --paired-end, --estimate-rspd, --calc -ci, mismatch rate = 0.3.

Variance-stabilised counts were used for principal component analysis (PCA) (10,000 most variable genes). Raw counts were used for DESeq2. The design formula included batch and stage. FPKM values were used exclusively for clustering and visualisation.

### 2.7 Differential expression analysis

Differentially expressed genes (DEGs) were identified with DESeq2 v1.26.0 (Love et al., 2014) with raw counts and the model: count ∼ batch + spermatogenic stage. Thresholds were |log_2_FC|≥1.5], |log_2_FC|≤−1.5 and *padj* ≤ 0.01 (BH-FDR).

Log_2_ FC values were shrunk using apeglm. Replicates were treated as independent biological replicates. All contrasts used SI as the baseline. One SI replicate showed partial clustering with SII/SIII but was retained after confirming its transition marker expression aligned with a prior sea bass study. This validated its inclusion as representative of early-stage biological variation (see Results). Full-dataset results are reported (Supplementary Table S3). Independent filtering was applied using DESeq2 defaults.

Stage I (SI) was selected as the reference condition because it represents the pre-meiotic, immature testis, prior to the appearance of spermatocytes and the onset of spermiogenesis, thereby providing a biologically appropriate baseline against which to quantify progressive transcriptional changes associated with spermatogenic maturation.

### 2.8 Functional enrichment analyses

Optimal number of expression clusters was determined via gap statistics using fviz_nbclust (*factoextra* v1.0.7). K-means clustering was applied on variance-stabilised expression counts of DEGs.

Functional enrichment was performed with ClusterProfiler v4.0 (Wu et al., 2021) and g:Profiler (g:GOSt; Kolberg et al., 2023) using human, zebrafish and European sea bass backgrounds) with *padj* ≤ 0.05.

Redundant GO terms were collapsed with the Markov Clustering Algorithm (MCL) using kappa similarity networks; representative terms were selected based on the highest −log_10_ (*padj*). KEGG enrichment and GeneCards-based gene function curation were incorporated (Supplementary Table S4).

### 2.9 Temporal expression patterns and orthology analysis

DEGs were classified into upregulated (log_2_FC ≥ 1.5; *padj* ≤ 0.001), downregulated (log_2_FC ≤ −1.5; *padj* ≤ 0.001), or not significant. A total of 132 dynamic expression trajectories were extracted (Supplementary Table S5) and visualised with easyalluvial v0.3.2 (Koneswarakantha, 2025).

Human-sea bass orthologues were retrieved using Ensembl BiomaRt v2.64.0 (Supplementary Table S6).

## 3 Results

### 3.1 Identification of spermatogenesis stages and cellular composition

**FIGURE 1:**
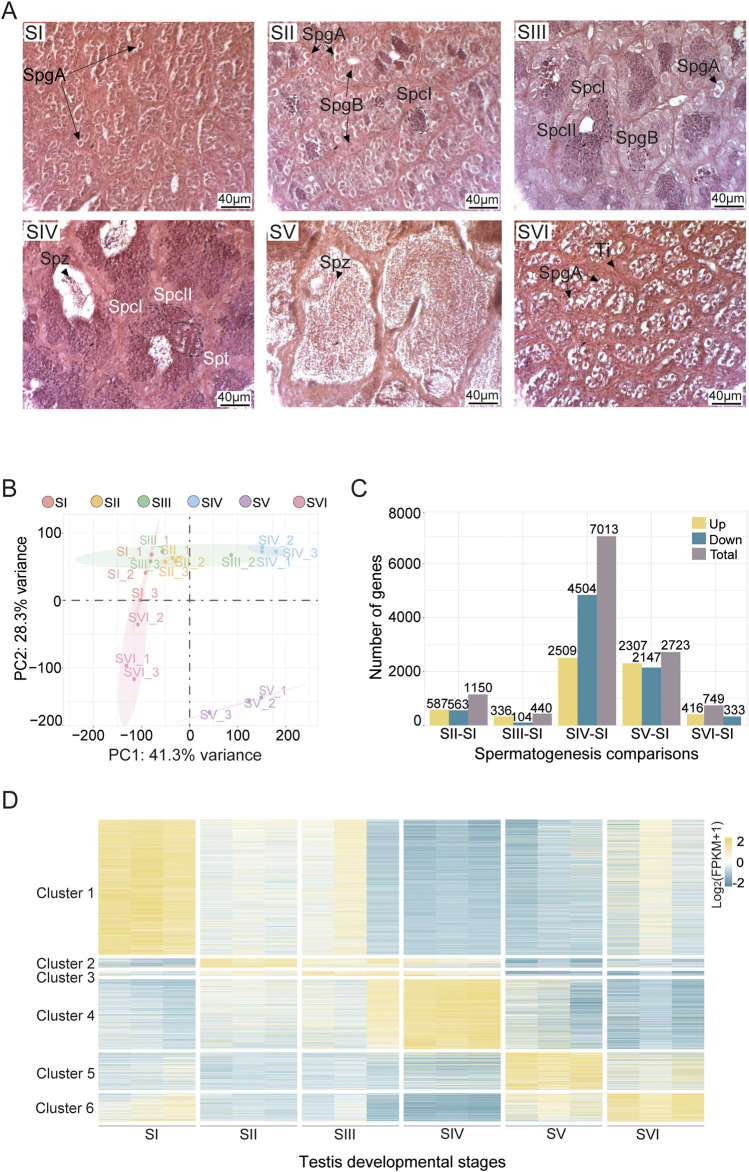
Histological staging, global transcriptomic structure, and gene expression dynamics across European sea bass spermatogenesis. **(A)** Representative haematoxylin-eosin-stained transverse testis sections illustrating the six histological stages of spermatogenesis (SI–SVI), following Begtashi et al. (2004). Annotated cell types include SpgA, SpgB, SpcI, SpcII, Sptd, Spz and interstitial cells (Ti). Scale bar: 40 μm. **(B)** PCA of the 10,000 most variable transcripts (variance-stabilised counts) showing stage-resolved clustering. PC1 (41.3%) reflects progression from SI-SIII to SIV-SV, and PC2 (28.3%) separates SVI. Ninety-five per cent confidence ellipses are shown. **(C)** Numbers of DEGs in each contrast relative to SI (DESeq2; |log_2_FC| ≥ 1.5; *padj* ≤ 0.01). Up- and downregulated genes are displayed separately. **(D)** Heatmap of DEGs grouped by K-means clustering (k = 6), ordered by peak stage. Colours represent row-scaled Z-scores of log_2_(FPKM + 1). Major transcriptional modules include hormone-responsive programmes, immune-associated transcripts, meiotic regulators, MAPK components, axonemal factors, and translational machinery.

European sea bass testes were sampled across six histological stages of the annual spermatogenic cycle (SI-SVI; first reproductive cycle). Representative haematoxylin-eosin micrographs illustrate the transition from proliferative spermatogonia in SI through early and mid recrudescence (SII-SIII), spermiogenesis (SIV), spermiation (SV), and post-spawning regression (SVI) (Figure 1A). Quantitative counts of SpgA, SpgB, SpcI, SpcII, Sptd, and Spz, respectively, cysts confirmed the expected shifts in germ-cell composition across stages (Supplementary Table S1).

During SI, lobules were dominated by SpgA (Supplementary Figure S1A). SII exhibited increasing numbers of SpgB and the first SpcI cysts, while SIII showed a higher abundance of SpcII, indicating progression through meiotic prophase. In SIV, spermatids and early spermatozoa became prominent, marking the onset of spermiogenesis. SV contained lobules filled with mature spermatozoa, and SVI displayed reorganisation of the seminiferous lobules structure together with a persistent SpgA pool (Figure 1A; Supplementary Figure S1A).

Among SI replicates, SI_1 contained a small SpgB population (Supplementary Figure S1B) and occupied an intermediate position between SI and SII/SIII in PCA space (Figure 1B). However, the expression of SI-informative markers (e.g., lower *cyp26a1* and *amh,* higher *pcna* and *hsf2bp*) in this replicate matched the SI classification profile stage (Supplementary Figure S1C). The heterogeneity in SI_1 did not reflect SII cellular heterogeneity and did not affect our conclusions.

### 3.2 Global transcriptome structure across spermatogenesis

PCA of the 10,000 most variable transcripts resolved a clear developmental trajectory (Figure 1B). PC1 (41.3%) captured the progression from SI–SIII to SIV-SV (spermiogenesis/spermiation), whereas PC2 (28.3%) separated SVI. All stages formed distinct clusters, although SI_1 and SIII_2 showed intermediate positions consistent with their histological features (Figure 1B). Top PCA gene loadings included *igf1, ccnd2a, il1b, tnfrsf1b, sycp3, hsf2bp, mapk11, ecm1b, aqp4* and *mgp* (Supplementary Figure S2A).

Differential expression analysis (DESeq2; |log_2_FC|≥1.5) revealed progressive transcriptomic divergence across stages (Figure 1C; Supplementary Table S3). The largest number of DEGs was detected at the onset of spermiogenesis (SIV), reflecting extensive changes in gene expression associated with haploid differentiation (Figure 1C).

K-means clustering (k = 6) grouped DEGs by stage-correlated expression patterns (Figure 1D; Supplementary Figure S2B). The six modules corresponded to: (i) *Cluster 1*: hormone-responsive and proliferative programmes (*igf1*, *ccnd2a*); (ii) *Cluster 2*: immune-associated transcripts (*il1b, tnfrsf1b*); (iii) *Cluster 3*: meiotic/centromeric regulators (*sycp3*, *hsf2bp*); (iv) *Cluster 4*: MAPK and signalling components (*mapk11*); (v) motility/axonemal factors (*dnah8, tuba*); and (vi) *Cluster 6*: translational machinery (e.g., *efl1*). Together, these clusters delineate the major transcriptional modules structuring spermatogenic progression.

### 3.3 Spermatogonial maintenance, endocrine regulation, and circadian signalling in the immature testis (SI)

SI exhibits high expression of canonical spermatogonial and early germline markers, including *id4*, *dnd1, piwil1*, *dmrt1,* and *tex14*, together with multiple piRNA pathway components (*tdrd1, tdrd12, ddx4*, *btbd18*) (Supplementary Figure S3A). SI included transcriptionally related genes, such as chromatin-associated and regulatory members, *brd1a*, *brdt, brd4*, *baz2b, mad1l1, kifc3, krt13* and *psme4* (Supplementary Figure S3A).

Functional enrichment of SI-downregulated genes (i.e., genes low at SI and increasing at later stages) identified categories related to hormone signalling responses, intercellular crosstalk, and cell-cell communication (Supplementary Figure S3B). Steroidogenic and endocrine-related transcripts, including *cyp17a1*, *star, gnrhr-1a*, *fshr, lhcgr, ghsra, esr2b*, *ar,* and *esrrb,* were also represented (Supplementary Figures S3A,B).

Components of circadian entrainment were detected at SI (Supplementary Figure S3C), including *per2, creb1, crebbpa, fosl2, fosb, c-fos, nos1, rasd1*, as well as glutamate receptors, voltage- and ligand-gated calcium channels, adenylate cyclases, ryanodine receptors, and CaMKII paralogues. The genes formed an interconnected signalling module converging on CREB (Supplementary Figure S3C).

### 3.4 Mitotic and meiotic regulation during early and mid-recrudescence (SII-SIII)

**FIGURE 2:**
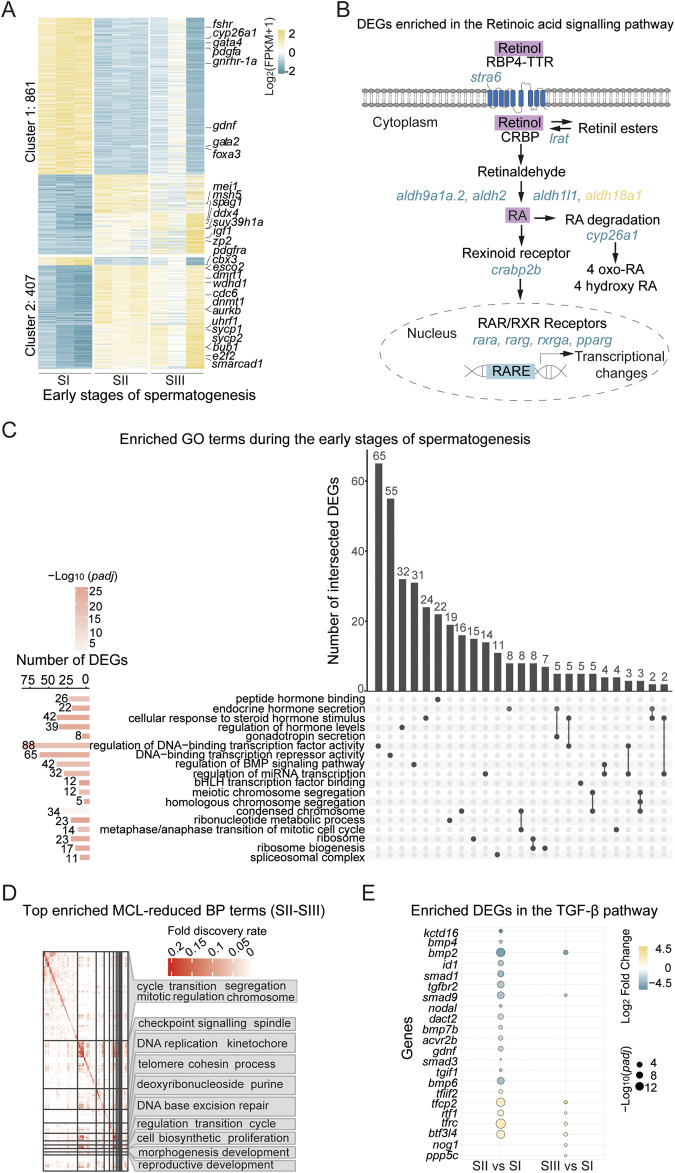
Transcriptomic transitions during early and mid-recrudescence (SII-SIII). **(A)** Heatmap of 1,267 DEGs in SII vs. SI and SIII vs. SI comparisons, grouped into two clusters enriched for endocrine/paracrine signalling, RA pathway components, meiotic regulators, and chromatin-associated genes. Values represent row-scaled Z-scores of log_2_(FPKM + 1). **(B)** Schematic representation of the RA pathway showing expression direction (yellow, upregulated; blue, downregulated; *padj* ≤ 0.01). Only genes passing significance thresholds are displayed. **(C)** UpSet plot of intersections among enriched GO terms across biological process (BP), molecular function (MF), and cellular component (CC) domains. **(D)** Enriched BP GO terms for SII vs. SI reduced by the MCL clustering, highlighting chromosome organisation, nuclear division, and DNA replication. **(E)** Dot plot of DEGs associated with TGF-ß/BMP signalling (*padj* ≤ 0.01), including ligands, receptors, intracellular mediators and regulatory components. Dot colour represents expression direction; size indicates significance (-log_10_ *padj*).

Comparisons of SII vs. SI and SIII vs. SI identified two main DEG clusters (Figure 2A). *Cluster 1* included endocrine and paracrine regulators (*fshr, gnrhr-1a, igf1, pdgfa/pdgfra*), retinoic acid (RA) pathway components (*cyp26a1, stra6, crabp2b*, *rara, rarg, rxrga*), meiotic recombination genes (*msh5*, *mei1*), and chromatin-associated regulators (*cbx3*, *uhrf1,* smarcad1a, *suv39h1a*). *Cluster 2* contained gene sets associated with chromosome segregation, kinetochore assembly, and meiotic prophase progression (Figure 2A).

The RA pathway diagram (Figure 2B) integrated the expression of RA transporters (*stra6, crabp2b*), nuclear receptors (*rara, rarg, rxrga, pparg*), aldehyde dehydrogenases (*aldh9a1a.2, aldh2, aldh1l1, aldh18a1*), and the RA-degrading enzyme *cyp26a1*. The combined expression patterns are shown for positional context, without mechanistic interpretation (Figure 2B).

Intersecting GO terms for SII and SIII (Figure 2C) indicated enrichment for hormone-related processes, bone morphogenetic protein (BMP) signalling, translation, chromosome dynamics, DNA replication, mitotic nuclear division, and sister-chromatid cohesion. MCL-reduced GO terms highlighted mitotic cell-cycle progression, chromosome organisation and segregation (Figure 2D; Supplementary Figure S4A).

KEGG pathway analysis identified enrichment in the transforming growth factor-beta (TGF-ß) and BMP signalling (Figure 2E; Supplementary Figure S4B). Upregulated members included ligands (*bmp2, bmp4, bmp6, bmp7*, *nodal*), receptors (*tgfbr2, acvr2b*), intracellular mediators (*smad1, smad3, smad9*), and additional pathway components (*dact2, id1, tgif1*) (Figure 2E; Supplementary Figure S4B).

### 3.5 Chromatin remodelling and flagellum assembly during spermiogenesis (SIV)

**FIGURE 3:**
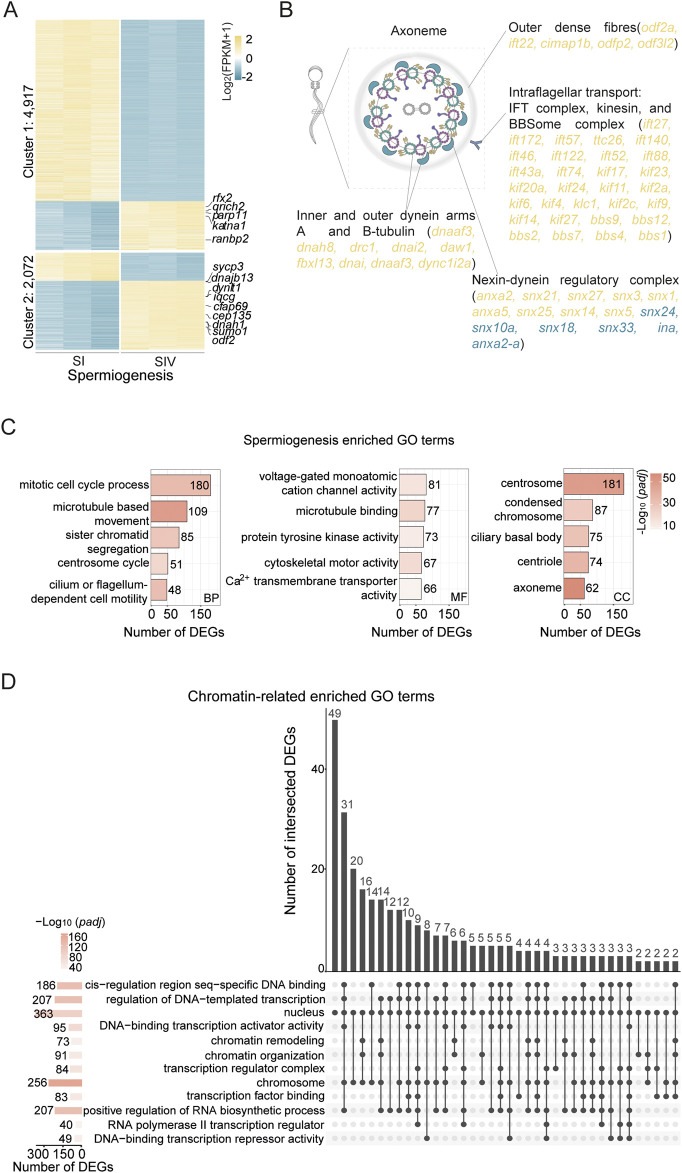
Molecular programmes associated with spermiogenesis (SIV). **(A)** Hierarchical k-means clustering of DEGs in SIV vs. SI identifies two major clusters enriched for fertility-associated genes. Colours represent row-scaled Z-scores of log_2_(FPKM + 1). **(B)** Schematic of the axoneme and sperm tail architecture, annotated with genes up- or downregulated at SIV (yellow, up; blue, down; *padj* ≤ 0.01). **(C)** Top enriched GO for DEGs in SIV vs. SI across BP, MF, and CC categories, highlighting processes related to centrosome cycle, microtubule-based movement, and axonemal organisation. **(D)** UpSet plot of 426 chromatin-associated DEGs, displaying intersections among genes linked to chromatin organisation, histone modification, heterochromatin assembly, and transcriptional regulation.

SIV corresponded to the onset of spermiogenesis, during which haploid spermatids undergo extensive morphological and molecular remodelling to form mature spermatozoa. SIV showed the largest transcriptional shift of the cycle, with 2,509 upregulated DEGs in the SIV vs. SI comparison (Figure 3A; Supplementary Table S3). Hierarchical clustering partitioned these DEGs into two major clusters: one enriched for genes associated with spermatid differentiation and the other for sperm structural components (Figure 3A). Both clusters contained fertility-related transcripts, including *rfx2*, *qrich2*, *parp11*, *katna1*, *ranbp2, sycp3, iqcg, cfap69, cep135, dnajb13, dynlt1, dnah1* and *cimap1b* (Supplementary Table S7).

The axoneme schematic (Figure 3B) illustrated a coordinated induction of genes coding for structural components involved in flagellum assembly, including inner and outer dynein arms, A/B-tubulin doublets, the nexin-dynein regulatory complex, the central pair apparatus, outer dense fibres (ODFs), and intraflagellar transport (IFT)/BBSome machinery (Figure 3B; Supplementary Figure S5A). Enriched GO terms for SIV vs. SI highlighted microtubule-based movement, axonemal organisation, centrosome cycle transitions, and cell division processes associated with late germ-cell maturation (Figure 3C; Supplementary Table S4).

Chromatin-related remodelling was extensive. A total of 426 chromatin-associated DEGs were detected at SIV (Supplementary Table S8), comprising histone variants (*h1, h1-1, h1-5, h2afv, h2az1*), numerous transcription factors, centrosomal and heterochromatin-associated genes (*cep135, plk1, nek2, odf2a, suv39h1a, smarcad1a, baz1a, rsf1*), and SUMO/E2-conjugating enzymes (*ube2s, ube2n, ube2r2, sumo1, sumo3l, ranbp2, znf451*) (Figure 3D; Supplementary Figure S5B). Thirty-six chromatin-related DEGs overlapped with proteins previously described in the European sea bass sperm proteome (Supplementary Table S8). Together, these transcriptomic features outline the main molecular dual programmes active during spermiogenesis: assembly of flagellar architecture and broad chromatin restructuring.

### 3.6 Sperm release, calcium signalling, and adhesion remodelling during spermiation (SV)

**FIGURE 4:**
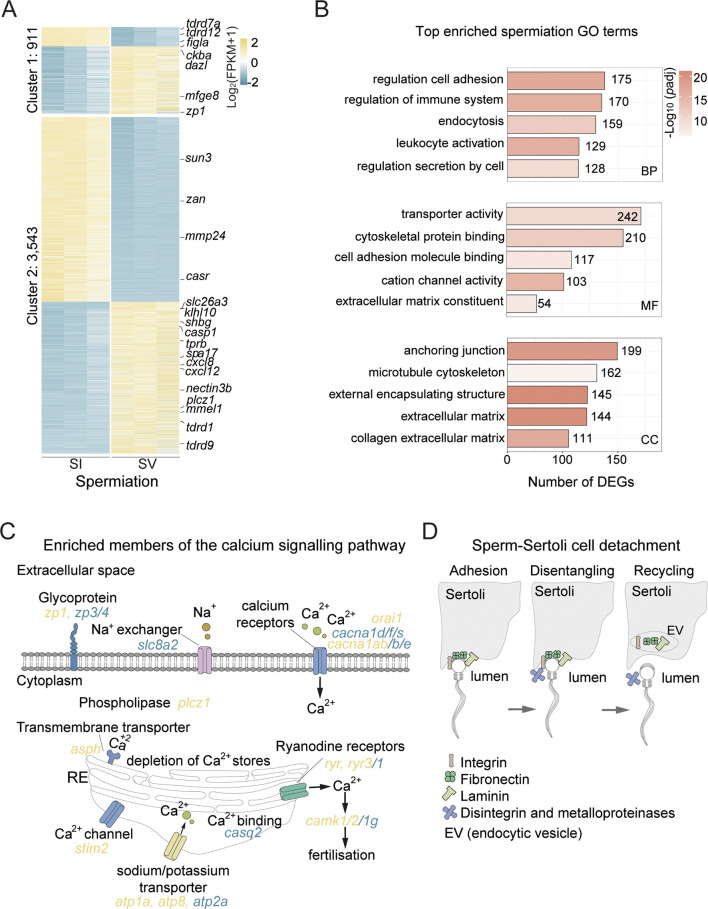
Transcriptomic features of spermiation (SV). **(A)** Heatmap of DEGs for SV vs. SI contrasts, separating two clusters enriched in differentiation-related and sperm-egg interaction genes. **(B)** Enriched GO categories associated with immune activity, leukocyte signalling and cell adhesion. **(C)** DEGs in the calcium signalling pathway (yellow, upregulated; blue, downregulated; *padj* ≤ 0.01), including voltage-gated Ca^2+^ channels, ryanodine receptors, and store-operated Ca^2+^ entry regulators. **(D)** Schematic of sperm-Sertoli cell attachment and integrin-mediated detachment, annotated with DEGs associated with laminins, integrins, disintegrins and ECM components.

SV, corresponding to spermiation, was characterised by the transcriptional enrichment of genes linked to sperm release and lobular remodelling. Hierarchical clustering identified two major DEG groups (Figure 4A). *Cluster 1* included glycoprotein-related and differentiation-associated genes such as *mfge8, zp1, figla, dazl, ckba*, whereas *Cluster 2* contained transcripts associated with sperm maturation and gamete interaction, including *spa17, plcz1, zan, mmel1, klhl10, sun3, tprb*, along with CXC chemokines (*cxcl8, cxcl10, cxcl12*) (Figure 4A).

GO enrichment analysis revealed processes associated with immune-system activity, leukocyte signalling, and regulation of cell adhesion (Figure 4B; Supplementary Figure S6A; Supplementary Table S4). DEGs enriched in the calcium-signalling pathway included voltage-gated calcium channels (*cacna1ab, cacna1b, cacna1e, cacna1d, cacna1f, cacna1s*), ryanodine receptors (*ryr1, ryr3*), and regulators of store-operated Ca^2+^ entry (*stim2, orai1*), as well as *plcz1* (Figure 4C). These genes were associated with Ca^2+^ influx and Ca^2+^ mobilisation (Figure 4C).

Graphic representation of adhesion components (Figure 4D) showed upregulated integrins (*itgb2, itgam, itgal, itga1, itga7, itga5*), fibronectin-related genes (*fn1, fndc11, fndc4*), laminins (*lama1, lama3*), and disintegrins/metalloproteinases (*adamts1, adamts1b, adamts2, adamts8, adamts15, adam22*). Downregulated elements included *itga6, lrfn2, lamc2, adamts6, adamts7, adamts10,* and *adamts18* (Figure 4D). Additional immunoglobulin- and cell-adhesion-molecule-related DEGs identified at this stage are depicted in Supplementary Figure S6B.

These transcriptomic features delineate the primary gene modules associated with sperm release, including Ca^2+^ signalling, extracellular matrix (ECM) remodelling and adhesion, and immune-related components active during spermiation.

### 3.7 Immune, apoptotic, and vascular processes during regression (SVI)

**FIGURE 5:**
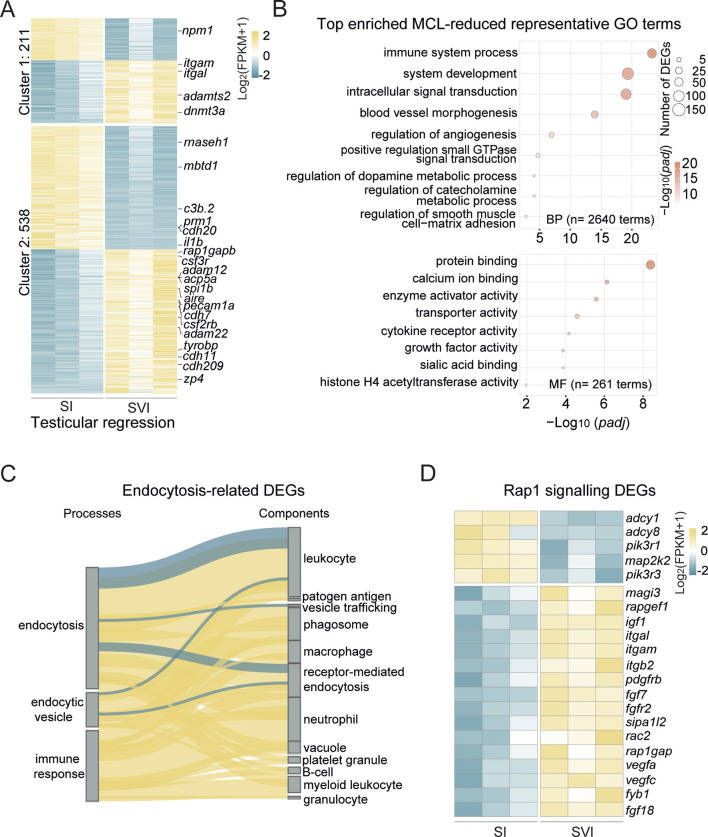
Regression (SVI): immune activation, phagocytosis, and vascular remodelling. **(A)** Heatmap of DEGs for SVI vs. SI showing clusters enriched in immune, vascular, and phagocytic processes. Values represent row-scaled Z-scores of log_2_(FPKM + 1). **(B)** Enriched GO BP and MF terms related to immune activation, intracellular signalling, and ECM remodelling. **(C)** Sankey plot of DEGs contributing to immune activation, endocytosis-mediated clearance, and adhesion turnover (yellow, upregulated; blue, downregulated; *padj* ≤ 0.01). **(D)** Heatmap of genes enriched in the Rap1-signalling pathway (*padj* ≤ 0.001), associated with vascular remodelling during regression. Values represent row-scaled Z-scores of log_2_(FPKM + 1).

During testicular regression, the interstitial compartment becomes enriched in somatic cells that actively contribute to tissue remodelling and re-establish the testicular framework required for the subsequent reproductive cycle. SVI showed transcriptional enrichment of genes involved in immune activity, phagocytosis, extracellular matrix turnover, and vascular remodelling (Figure 5A). Hierarchical clustering of SVI vs. SI DEGs revealed two main groups: one enriched for vasculature- and phagocytosis-related transcripts, and the other for immune-response genes (Figure 5A; Supplementary Table S3).

Genes associated with immune activation included *spi1b, tyrobp, acp5, csf3r, cd209* and *il1b*, while ECM-related and adhesion components (*adamts2, cdh7, cdh11*) were also upregulated. Six immune-related DEGs (*il1b, mx, c3b.2, dic, h1, h2b*) corresponded to previously reported components of the European sea bass immune response (Supplementary Table S3). Vascular and angiogenesis genes (*vegfa, vegfc, fgf7, fgfr2, rap1gapb*) were detected within the same stage-specific cluster (Figure 5A).

GO enrichment analysis highlighted categories associated with immune activation, intracellular signalling, blood vessel morphogenesis, and extracellular matrix organisation (Figure 5B; Supplementary Table S4). Sankey diagrams showed the contributions of DEGs to immune pathways, phagocytic uptake, adhesion, and ECM dynamics (Figure 5C). KEGG analysis showed enrichment for Rap1-related signalling (Figure 5D), which included several genes involved in endothelial and cell-matrix remodelling (Figure 5D).

### 3.8 Dynamic gene expression patterns across the spermatogenic cycle

**FIGURE 6:**
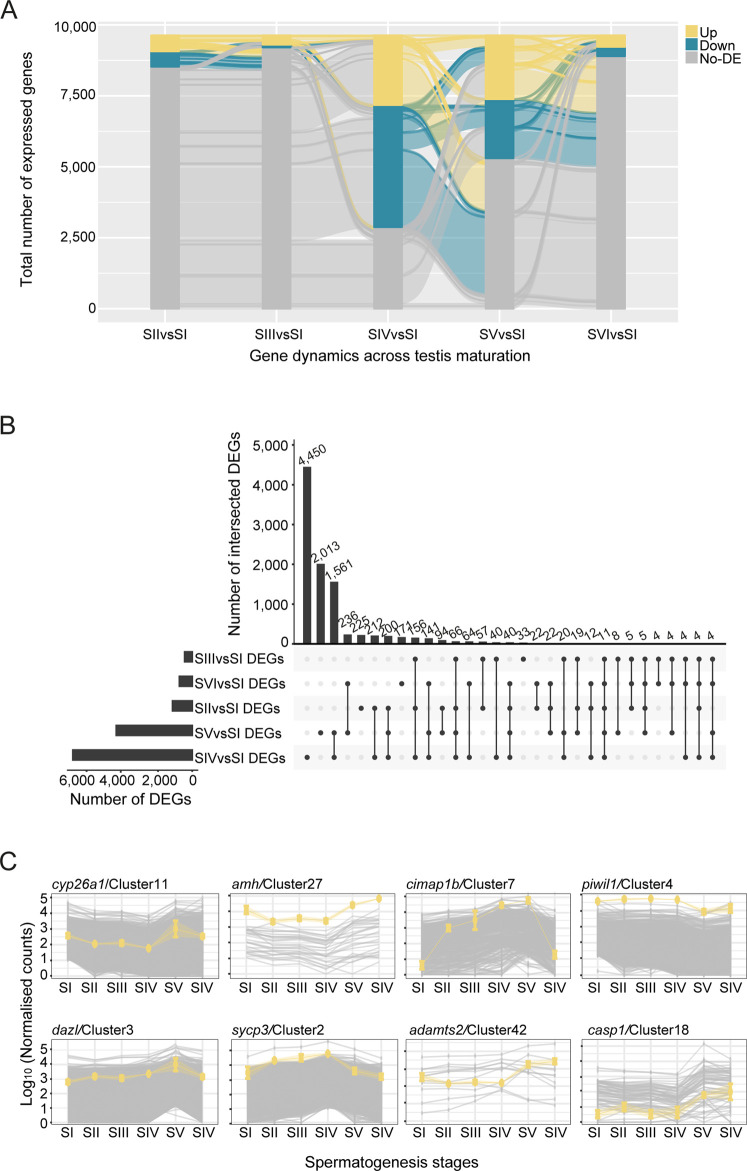
Dynamic gene expression patterns across the spermatogenic cycle. **(A)** Alluvial plot of 9,327 DEGs showing dynamic transcriptional trajectories across SI-SVI. **(B)** UpSet plot showing intersections of DEGs between SIV vs. SI and SV vs. SI. Bar heights represent the number of DEGs in each intersection; connected dots below specify which pairwise comparisons contribute to each set. **(C)** Representative expression profiles of stage-informative markers across the cycle.

To examine transcriptional dynamics across the full annual cycle, we analysed the trajectories of 9,327 DEGs across all stages (Figure 6A; Supplementary Table S5). Alluvial visualisation resolved 132 discrete temporal profiles, identifying stage-restricted and stage-persistent gene-expression patterns (Figure 6A). This trajectory-based analysis integrates sequential transitions between adjacent stages and captures transient and stage-restricted expression dynamics that may not be fully resolved by baseline-referenced contrasts alone.

A major group of genes showed SIV-restricted activation, being low at SI-SIII, strongly upregulated during SIV and returning to baseline at SV-SVI. This set (1,660 genes) included many axonemal, motility, and chromatin-associated factors represented in Figures 3, 6A, and in Supplementary Figure S5, together with genes listed in the fertility-related set (Supplementary Table S7).

Another large group exhibited SV-restricted upregulation (1,060 genes), matching categories associated with adhesion, immune-related activity, calcium signalling and ECM remodelling (Figures 4, 6A; Supplementary Figure S6).

Genes with shared SII-III upregulation formed a coherent early-recrudescence pattern, showing increased expression from SII to SIV relative to SI (Figure 6B). These included regulators of chromosome segregation and mitotic control, such as *zwint, ccnf, cdk1, fbxo5, knl1, tacc3,* and *nsl1* (Figures 2C–E; Supplementary Figures S4A, S7).

In contrast, many spermiogenesis-associated genes displayed SIV-to-SV expression peaks (*sumo1*, *ccdc63, ift27, ccdc65*, *septin7, tekt1, spag8*, *gas8*, and numerous cilia and flagella-associated proteins (CFAP) family members) (Figure 3B; Supplementary Figures S6A–7B). Ate-stage genes (*klhl10, psmc4, psmb7, cacna1i, cacna1ab, plcz1, nectin3b*) showed SIV-SV activation patterns (Figures 4A,C; Supplementary Figure S7).

Additional trajectory patterns included early SI-skewed expression (*cd33*, *aif1*) decreasing through SIV (Supplementary Figures S3, S7) and late SV-SVI induction of immune and remodelling genes (*rel, itgal, aif1, cd209, cd22, casp8, casp1, mmp14b*) (Figures 4A, 5A, 6B; Supplementary Figures S6, S7).

Temporal expression profiles of representative markers (Figure 6C) captured these patterns. *Cyp26a1* showed early downregulation with later re-expression; *amh* decreased after SI and increased at later stages; *cimap1b* displayed SIV-associated activation; *piwil1* maintained expression from SI; *sycp3* peaked at SIV; *adamts2* increased during SV-SVI; and *casp1* rose at late stages (Figure 6C).

## 4 Discussion

### 4.1 An integrated transcriptional roadmap of seasonal spermatogenesis

By integrating histological staging with transcriptomic profiling across the six spermatogenic stages (SI-SVI), this study provides a comprehensive view of annual testicular maturation in European sea bass. The dataset resolves major biological transitions, including spermatogonial maintenance, meiotic licensing, spermiogenesis, spermiation, and regression. These results expand on the cellular descriptions previously reported for teleost testes (Schulz et al., 2010; Schulz et al., 2023; Uribe et al., 2014) and complement transcriptomic resources available for other seasonal or marine species (Jin et al., 2024; Wang X. et al., 2023; Zhang et al., 2016; Roy et al., 2017; Luo et al., 2019; Castro-Arnau et al., 2022).

### 4.2 Endocrine, paracrine, and circadian components at spermatogenic onset

The onset of spermatogenesis in European sea bass is characterised by the convergence of endocrine, paracrine, and circadian transcriptional programmes within the immature testis. At Stage I, the coordinated expression of gonadotropin receptors, steroid-responsive elements, and growth-factor signalling components, together with circadian regulators converging on CREB-dependent pathways, defines a regulatory environment permissive for spermatogonial activation. The presence of *gnrhr-1a, fshr, lhcgr, ghsra, esr2b, ar,* and *igf1,* together with clock-associated genes, such as *creb1, crebbpa, per2, fosl2, fosb, c-fos* and *rasd1*, is consistent with previous evidence that photoperiodic cues, endocrine signalling and local paracrine factors act in concert to regulate the onset of spermatogenesis in European sea bass and other teleosts (Martins et al., 2015; Espigares et al., 2017; Blázquez et al., 2017; Alvarado et al., 2024). In addition, the expression of modulators of early spermatogenic activity, including components of the TGF-ß/BMP axis and factors such as *gdnf* and *amh,* agrees with their established roles in regulating spermatogonial maintenance and differentiation in fish testes (Picha et al., 2008; Bellaïche et al., 2014; Pfennig et al., 2015; Crespo et al., 2016; Mohammed-Geba et al., 2016; Blázquez et al., 2017; Fernandes da Costa et al., 2024). Together, these data support a model in which spermatogonial recruitment emerges from the alignment of systemic hormonal inputs with locally gated circadian transcriptional control rather than from a single dominant regulatory signal.

### 4.3 Regulatory signatures associated with meiotic licensing during recrudescence

The transition between SII and SIII is characterised by the coordinated upregulation of genes associated with DNA replication, chromosome organisation, kinetochore assembly, and recombination, together with chromatin and genome-stability regulators, such as *uhrf1, smarcad1a,* and *suv39h1a*, and recombination-associated genes including *msh5* and *mei1*. This transcriptional configuration delineates a regulatory checkpoint preceding meiotic entry and reflects regulatory themes consistent with the establishment of meiotic competence described in comparative vertebrate studies (Hirano, 2012; Rankin, 2015). In parallel, the induction of retinoic-acid-associated components (*stra6, crabp2b, rara, rarg, rxrga, aldh9a1a.2, aldh1l1, aldh18a1,* and *cyp26a1*) coincides with RA-dependent signatures of meiotic licensing reported in teleosts and other vertebrates (Miura and Miura, 2003; Adolfi et al., 2016; Blázquez et al., 2017; Skaar et al., 2011; Crespo et al., 2019; Medina et al., 2019). Together, these signatures indicate that meiotic competence during recrudescence is established through a progressive regulatory state that integrates retinoic acid signalling with chromatin-regulatory and epigenetic modules, genome-stability control, and checkpoint coordination.

### 4.4 Spermiogenesis as a dual programme of motility and chromatin remodelling

Spermiogenesis constitutes the largest transcriptional shift of the cycle, coinciding with the completion of meiosis and the onset of haploid differentiation. What emerges is a highly integrated programme in which cytoskeletal expansion and nuclear condensation unfold in parallel. SIV shows a strong activation of axonemal motors, IFT/BBSome components, dynein and tubulin subunits, nexin-dynein regulatory complex members and ODFs, all signs of the structural demands of flagellum assembly and with conserved mechanisms observed in fish and mammals (Moore et al., 2013; Ben Khelifa et al., 2014; Prokopchuk et al., 2017).

In parallel, SIV induced a broad induction of chromatin-associated regulators, including histone variants, transcription factors, heterochromatin-linked genes, and SUMO/E2-conjugating enzymes, consistent with previously documented chromatin restructuring during spermatid maturation. Several of these SIV-induced factors overlap with proteins previously identified in the European sea bass sperm proteome (Barrachina et al., 2018).

Spermiogenesis, therefore, emerges as a tightly coordinated dual programmes in which cytoskeletal expansion and nuclear remodelling proceed in parallel. The temporal coupling of axonemal assembly and chromatin restructuring indicates that post-meiotic differentiation is governed by interconnected regulatory modules rather than by independent structural and nuclear processes.

### 4.5 Spermiation: adhesion turnover, ECM dynamics, and Ca^2+^ signalling

Spermiation emerges as a complex remodelling event characterised by enrichment of adhesion molecules, cytoskeletal regulators, immunoglobulin-related transcripts, integrins, laminins, and metalloproteinases. Although our transcriptomic data cannot assign causality, the upregulation of calcium-associated components, including *plcz1*, ryanodine receptors, voltage-gated calcium channels*, stim2*, and *orai1*, are consistent with Ca^2+^-associated processes known to occur during gamete activation in vertebrates (Belardin et al., 2019; Jimenez-Gonzalez et al., 2006). The expression patterns observed here correspond to ECM restructuring, junctional remodelling and signalling elements previously described during spermiation in teleosts (Wang H-Y. et al., 2023; Yang et al., 2024).

The transcriptional enrichment of calcium-associated signalling components, adhesion molecules, and extracellular-matrix remodelling factors indicates that spermiation is an active, regulated process rather than a passive release of sperm. The induction of voltage-gated calcium channels, ryanodine receptors, *stim2*, *orai1*, and *plcz1*, together with integrins, laminins, and metalloproteinases, is consistent with Ca^2+^-dependent signalling and junctional remodelling mechanisms previously described during sperm activation and release in vertebrates (Jimenez-Gonzalez et al., 2006; Belardin et al., 2019). Similar transcriptional signatures associated with extracellular matrix turnover and Sertoli-germ cell detachment have been reported in teleost spermiation (Valero et al., 2015; Wang H-Y. et al., 2023; Yang et al., 2024), supporting a model in which ionic signalling and structural remodelling are tightly coupled at this stage.

### 4.6 Regression as an active phase of clearance and tissue reorganisation

Rather than representing a passive post-spawning state, testicular regression in European sea bass is characterised by the activation of immune, phagocytic, and vascular-remodelling transcriptional programmes. The induction of leukocyte-associated genes (*spi1b*, *tyrobp, acp5, csf3r, cd209*), together with ECM-related factors and angiogenic regulators (*vegfa, vegfc, fgf7, fgfr2, rap1gapb*), is consistent with immune-mediated clearance and tissue restructuring processes described in seasonal teleosts (Grier and Taylor, 1998; Chaves-Pozo et al., 2005; Valero et al., 2015; Ribeiro et al., 2017).

The concurrent persistence of spermatogonia and the expression of epigenetic regulators such as *dnmt3a* is consistent with mammalian models in which regression simultaneously removes differentiated germ cells while preserving a germline reservoir for the subsequent reproductive cycle (Dura et al., 2022).

### 4.7 Temporal dynamics and stage-informative markers

The 132 temporal trajectories captured stage-restricted peaks (e.g., SIV-exclusive, SV-exclusive) as well as persistent patterns (SI-high, SII-SIII, SIV-SV, SVI-late), consistent with the temporal logic observed in other teleosts (Roy et al., 2017; Zhang et al., 2016). Representative markers, *amh, piwil1, cyp26a, sycp3, cimap1b, adamts2* and *casp1*, provided consistent stage-correlated profiles. These markers complement previously described regulators of gonadal development in European sea bass and related species (Viñas and Piferrer, 2008; Crespo et al., 2013; Ribas et al., 2019).

The temporal trajectories resolved in this study identify a set of stage-informative genes that constitute candidate operational markers of spermatogenic progression in European sea bass. These markers allow discrimination between immature, recrudescent, spermiogenic, and regressing testes based on molecular signatures rather than chronological age alone, which is particularly relevant given the asynchronous nature of sexual maturation in this species. In an aquaculture context, such markers may be used diagnostically to detect the onset of spermatogenesis and to classify individual males into early, intermediate, or advanced stages of spermatogenesis. Although the present work does not address their implementation under farming conditions, these candidate operational markers provide a molecular framework for monitoring reproductive status and identifying precociously maturing males, thereby informing broodstock management strategies. Validation and application in commercial settings will require targeted follow-up studies under controlled production conditions.

Taken together, these stage-specific transcriptional programmes delineate a coherent regulatory framework in which endocrine, chromatin, cytoskeletal, calcium-signalling and immune-associated modules are sequentially deployed to drive spermatogenic progression across the annual reproductive cycle.

### 4.8 Limitations and future directions

Although this study provides a comprehensive, stage-resolved transcriptomic atlas of European sea bass spermatogenesis, several considerations should be acknowledged when interpreting the findings. The use of bulk RNA-seq provides a robust overview of the molecular landscape across the annual reproductive cycle; however, it does not resolve transcriptional contributions from specific germ or somatic cell populations. As demonstrated in zebrafish using single-cell and spatial transcriptomic approaches (Qian et al., 2022; Burgos-Ruíz et al., 2025), higher-resolution methods would enable future studies to disentangle cell-type-specific regulatory programmes and refine understanding of lineage interactions throughout the cycle.

In addition, while the dataset identifies numerous candidate regulators, particularly those associated with chromatin dynamics, meiotic progression, and flagellar assembly, the work does not include functional validation. Targeted experiments and stage-resolved histological confirmation would be valuable for determining the causal roles of these candidates and clarifying their mechanistic contribution to germ cell development.

A further limitation concerns the relationship between transcript abundance and protein-level activity. Although some SIV-associated transcripts overlap with proteins previously detected in the European sea bass sperm proteome (Barrachina et al., 2018), proteomic characterisation across all stages of the cycle would provide a more complete picture of translational regulation, post-translational modification, and protein turnover during spermatogenesis.

Finally, it is important to consider the study’s environmental context. Spermatogenesis in European sea bass is strongly influenced by temperature and photoperiod (Díaz and Piferrer, 2015; Zanuy et al., 2001), and the transcriptional dynamics described here were obtained under controlled conditions. Investigating how the identified genes and pathways respond to environmental modulation would help determine the plasticity of these molecular programmes and their relevance to aquaculture settings, where precocious maturation and altered reproductive timing remain major challenges.

## 5 Conclusion

This study presents the first stage-resolved transcriptomic atlas of spermatogenesis in European sea bass, integrating detailed histological characterisation with gene expression profiling across the six canonical stages of the annual reproductive cycle. The dataset reveals that the onset of spermatogenesis involves the coordinated activity of endocrine, circadian, and paracrine signalling pathways, which collectively shape the early spermatogonial environment. During recrudescence, transcriptomic signatures associated with RA metabolism, chromosome organisation, DNA replication and genome-stability pathways define the establishment of meiotic competence.

The onset of spermiogenesis marks the major transcriptional transition, with the parallel activation of programmes responsible for flagellar cytoskeletal assembly and extensive chromatin remodelling. Subsequent stages, spermiation and regression, are not transcriptionally quiescent but instead display dynamic changes involving the turnover of adhesion complexes, ECM remodelling, calcium-associated signalling and immune-driven tissue reorganisation. Together, these demonstrate that the European sea bass reproductive cycle unfolds as a tightly orchestrated sequence of molecular transitions that reflect the underlying cellular events observed histologically.

The identification of robust, stage-resolved molecular signatures yields a set of candidate operational markers for characterising spermatogenic status in European sea bass. These markers provide a molecular tool to monitor the progression of testicular maturation and to distinguish among early, mid-, and late spermatogenic stages in individual males, independently of age. While their practical deployment in aquaculture systems remains to be validated, this resource establishes a foundation for developing diagnostic approaches to identify precocious maturation and support informed broodstock management. Beyond its applied relevance, the atlas constitutes a comparative framework for vertebrate spermatogenesis and a reference for future studies investigating the molecular and epigenetic regulation of germ-cell development in seasonal species.

## Data Availability

The datasets presented in this study can be found in online repositories. The names of the repository/repositories and accession number(s) can be found in the article/Supplementary Material.
