# Supplementary material for: An integrated roadmap of European sea bass (Dicentrarchus labrax) spermatogenesis across the annual reproductive cycle
Source: Front Cell Dev Biol. 2026 Jun 24;14:1852477. doi: 10.3389/fcell.2026.1852477 (PMC13342237; doi:10.3389/fcell.2026.1852477)
Supplement: Supplementary file 3 [file Image5.pdf]

Supplementary Figure 5

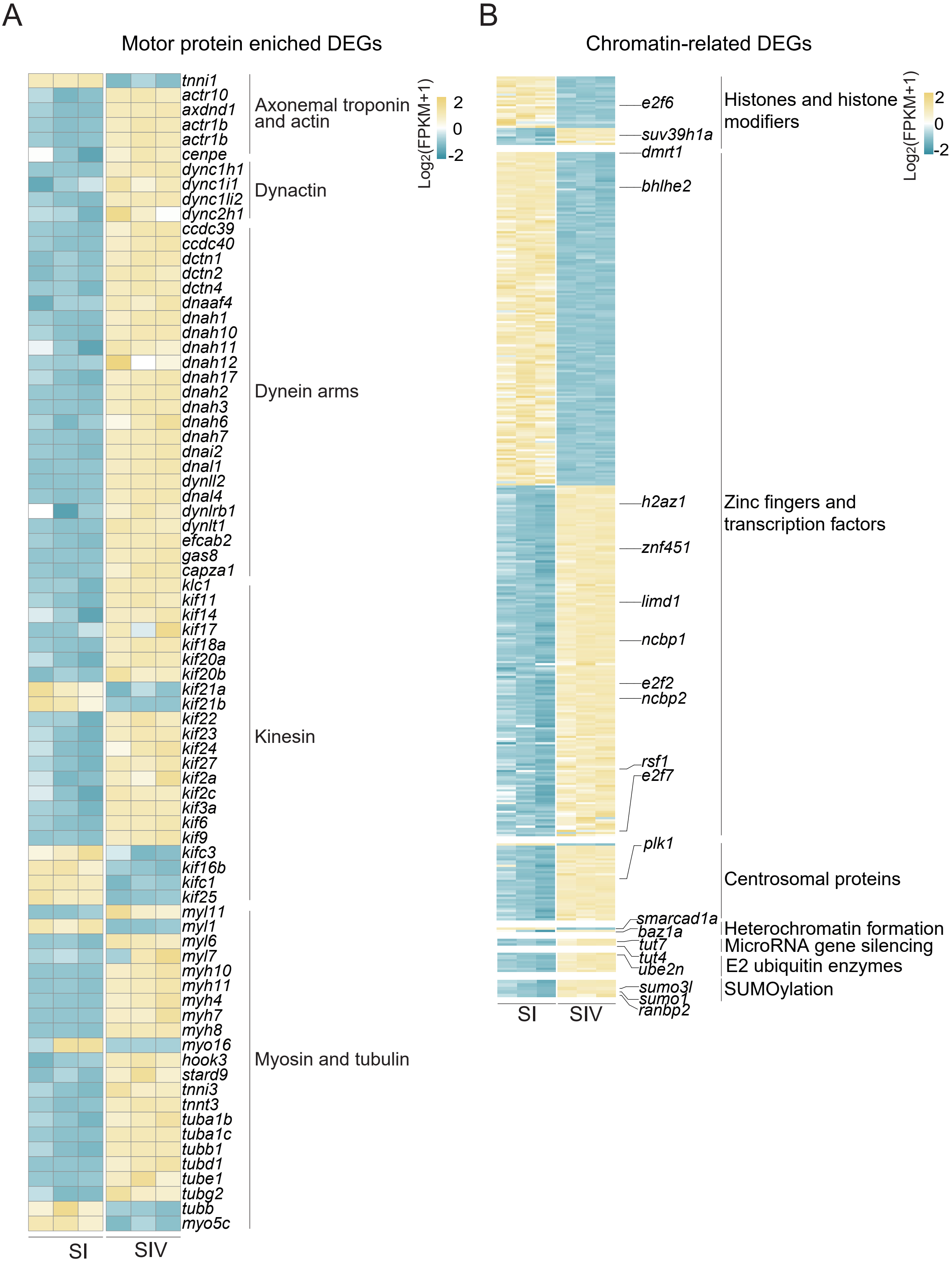

Supplementary Figure 5. Motor protein and chromatin-related DEGs during SIV. **(A)** Heatmap of DEGs enriched in motor protein pathways. Values represent row scaled Z scores of $\log_2$(FPKM + 1). **(B)** Chromatin-related DEGs span histone variants, transcription factors, and post-transcriptional regulators. Values represent row scaled Z scores of $\log_2$(FPKM + 1).
