# Supplementary material for: An integrated roadmap of European sea bass (Dicentrarchus labrax) spermatogenesis across the annual reproductive cycle
Source: Front Cell Dev Biol. 2026 Jun 24;14:1852477. doi: 10.3389/fcell.2026.1852477 (PMC13342237; doi:10.3389/fcell.2026.1852477)
Supplement: Supplementary file 4 [file Image6.pdf]

Supplementary Figure 6

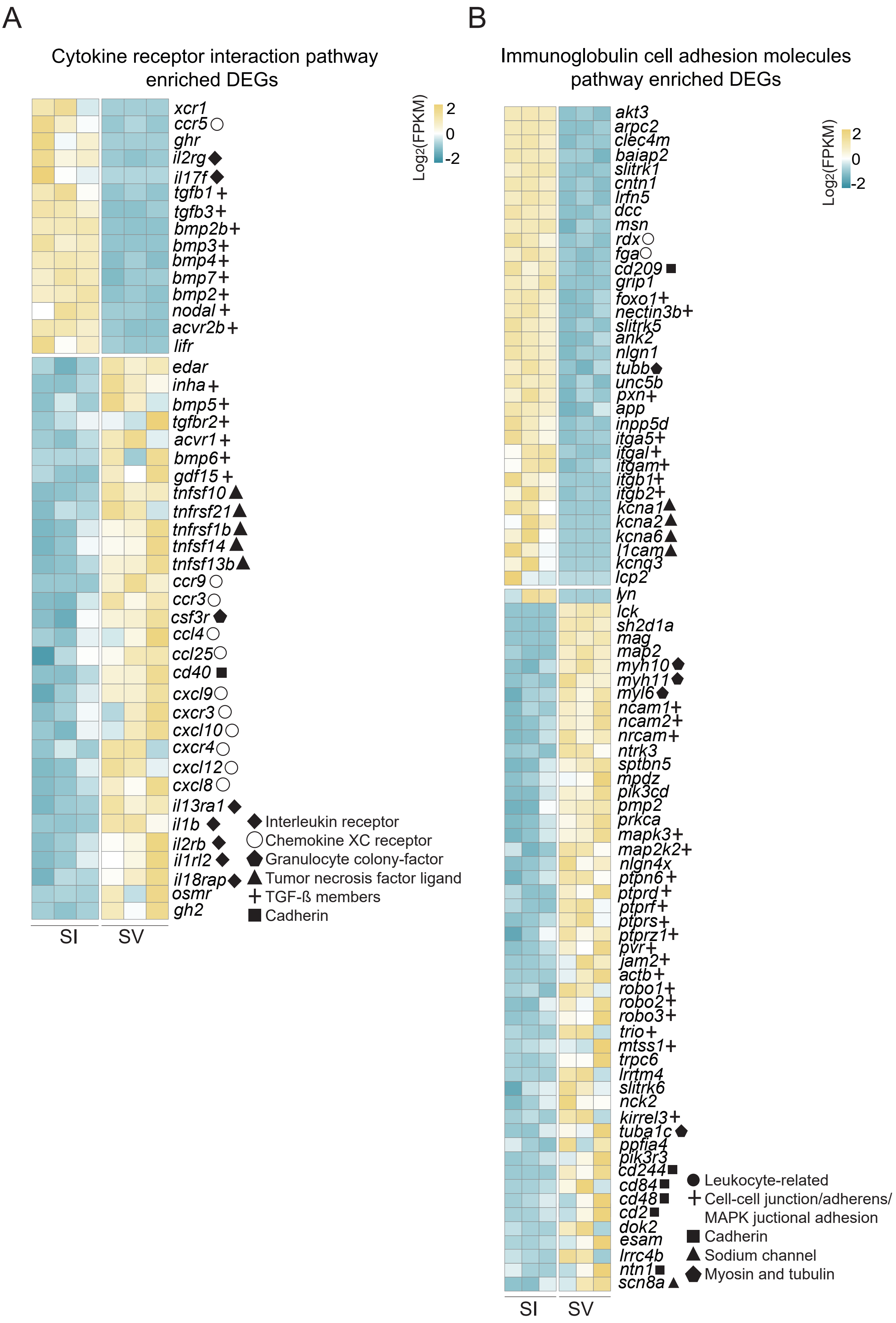

Supplementary Figure 6. Immune activation, adhesion remodelling and cytokine signalling during SV. **(A)** Heatmap of DEGs in the cytokine-cytokine receptor pathway. Values represent row scaled Z scores of $\log_2$(FPKM + 1). **(B)** DEGs associated with cell adhesion molecules, immunoglobulin-like proteins, and ECM-related components. Values represent row scaled Z scores of $\log_2$(FPKM + 1).
