# Supplementary material for: An integrated roadmap of European sea bass (Dicentrarchus labrax) spermatogenesis across the annual reproductive cycle
Source: Front Cell Dev Biol. 2026 Jun 24;14:1852477. doi: 10.3389/fcell.2026.1852477 (PMC13342237; doi:10.3389/fcell.2026.1852477)
Supplement: Supplementary file 5 [file Image4.pdf]

Supplementary Figure 4

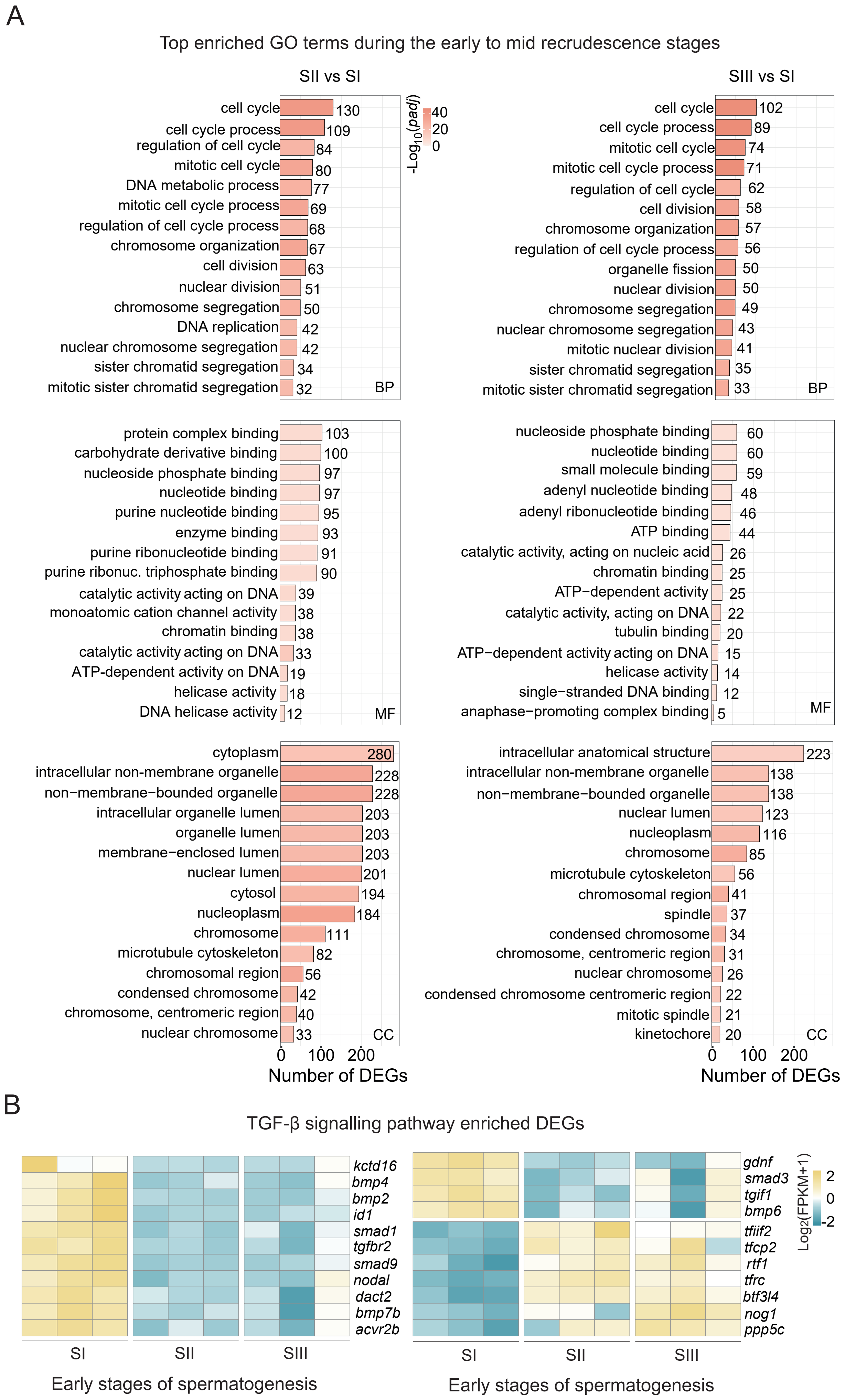

Supplementary Figure 4. GO and KEGG enrichment during SII-SIII. (**A**) Enriched GO terms for SII vs SI and SIII vs SI across BP, MF, and CC categories. (**B**) Heatmap of TGF-ß pathway components. Values represent row scaled Z scores of $\log_2$(FPKM + 1).
