# Supplementary material for: An integrated roadmap of European sea bass (Dicentrarchus labrax) spermatogenesis across the annual reproductive cycle
Source: Front Cell Dev Biol. 2026 Jun 24;14:1852477. doi: 10.3389/fcell.2026.1852477 (PMC13342237; doi:10.3389/fcell.2026.1852477)
Supplement: Supplementary file 6 [file Image2.pdf]

Supplementary Figure 2

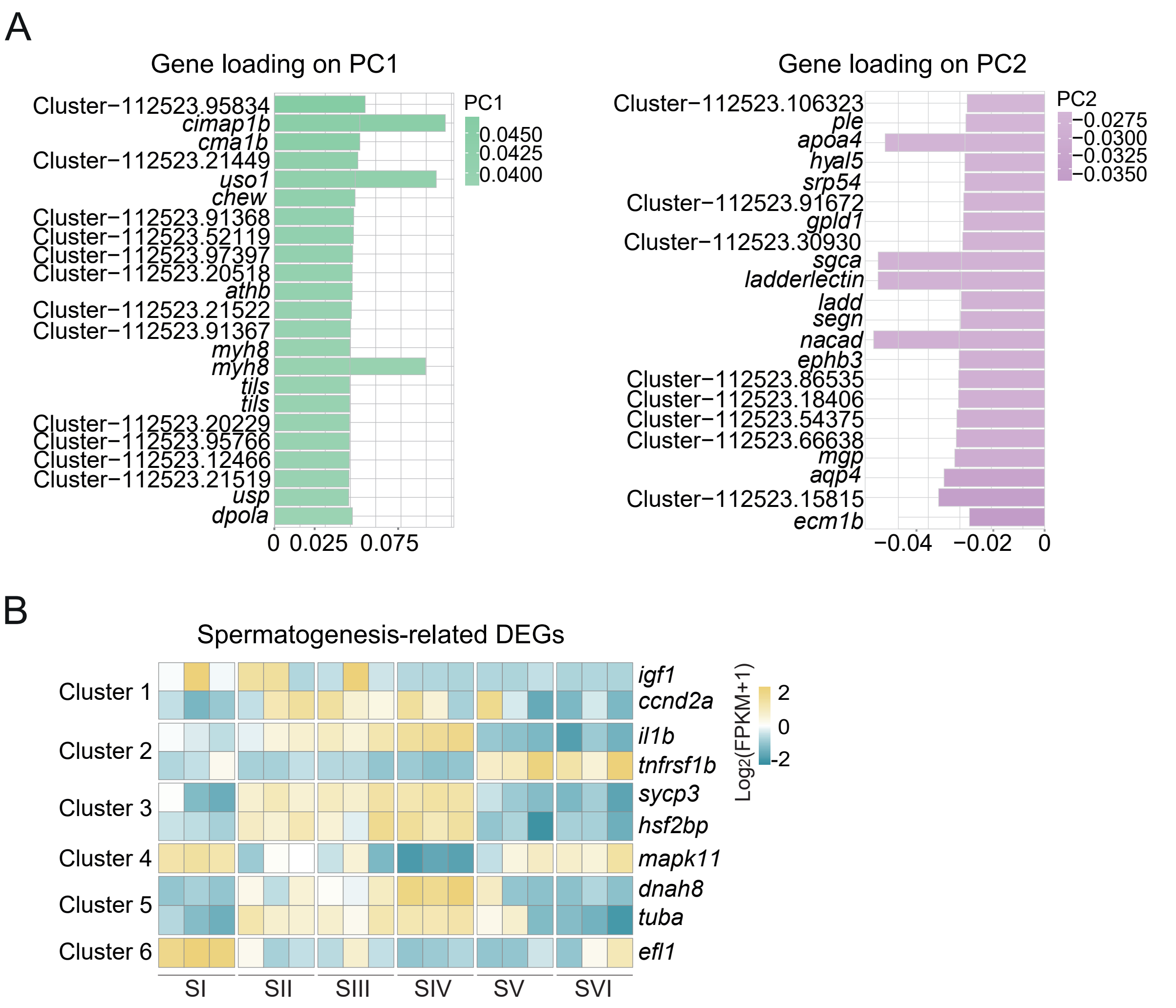

Supplementary Figure 2. PCA drivers and representative cluster markers. **(A)**Top 20 PCA loading for PC1 and PC2 among the 10,000 most variable transcripts. Bar length is proportional to absolute loading; direction is indicated by bar colour. **(B)** Representative DEGs for each k-means cluster (1-6). Values represent row scaled Z-scores of $\log_2$(FPKM+1).

Supplementary Figure 2. PCA drivers and representative cluster markers. **(A)**Top 20 PCA loading for PC1 and PC2 among the 10,000 most variable transcripts. Bar length is proportional to absolute loading; direction is indicated by bar colour. **(B)** Representative DEGs for each k-means cluster (1-6). Values represent row scaled Z-scores of log_2(FPKM+1).

Supplementary Figure 2. PCA drivers and representative cluster markers. **(A)**Top 20 PCA loading for PC1 and PC2 among the 10,000 most variable transcripts. Bar length is proportional to absolute loading; direction is indicated by bar colour. **(B)** Representative DEGs for each k-means cluster (1-6). Values represent row scaled Z-scores of log_2(FPKM+1).
