# Supplementary material for: An integrated roadmap of European sea bass (Dicentrarchus labrax) spermatogenesis across the annual reproductive cycle
Source: Front Cell Dev Biol. 2026 Jun 24;14:1852477. doi: 10.3389/fcell.2026.1852477 (PMC13342237; doi:10.3389/fcell.2026.1852477)
Supplement: Supplementary file 8 [file Image3.pdf]

Supplementary Figure 3

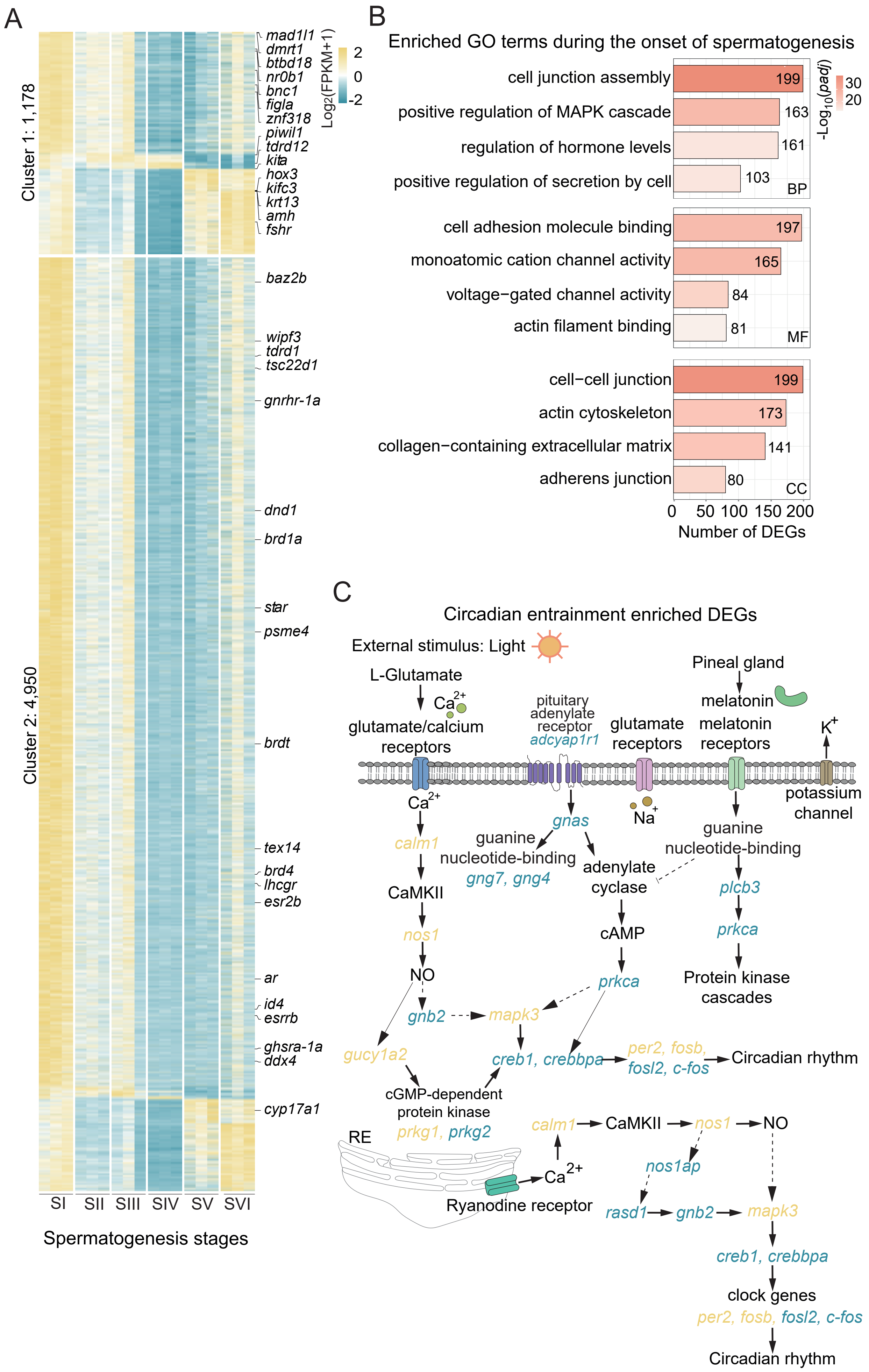

Supplementary Figure 3. SI-associated transcriptional profiles. **(A)** Heatmap of 6,128 SI-downregulated DEGs, including piRNA pathway genes and transcriptional regulators. Values represent row scaled Z scores of $\log_2(\text{FPKM} + 1)$. **(B)** Top GO-enriched terms in SI-downregulated genes. **(C)** DEGs in the circadian entrainment pathway showing multiple signalling cascades converging on CREB (yellow, upregulated; blue, downregulated; padj≤0.01). Solid arrows indicate activation; dashed arrows, indirect or unknown influences; dashed lines, inhibition.
