# Supplementary material for: An integrated roadmap of European sea bass (Dicentrarchus labrax) spermatogenesis across the annual reproductive cycle
Source: Front Cell Dev Biol. 2026 Jun 24;14:1852477. doi: 10.3389/fcell.2026.1852477 (PMC13342237; doi:10.3389/fcell.2026.1852477)
Supplement: Supplementary file 11 [file Image7.pdf]

Supplementary Figure 7

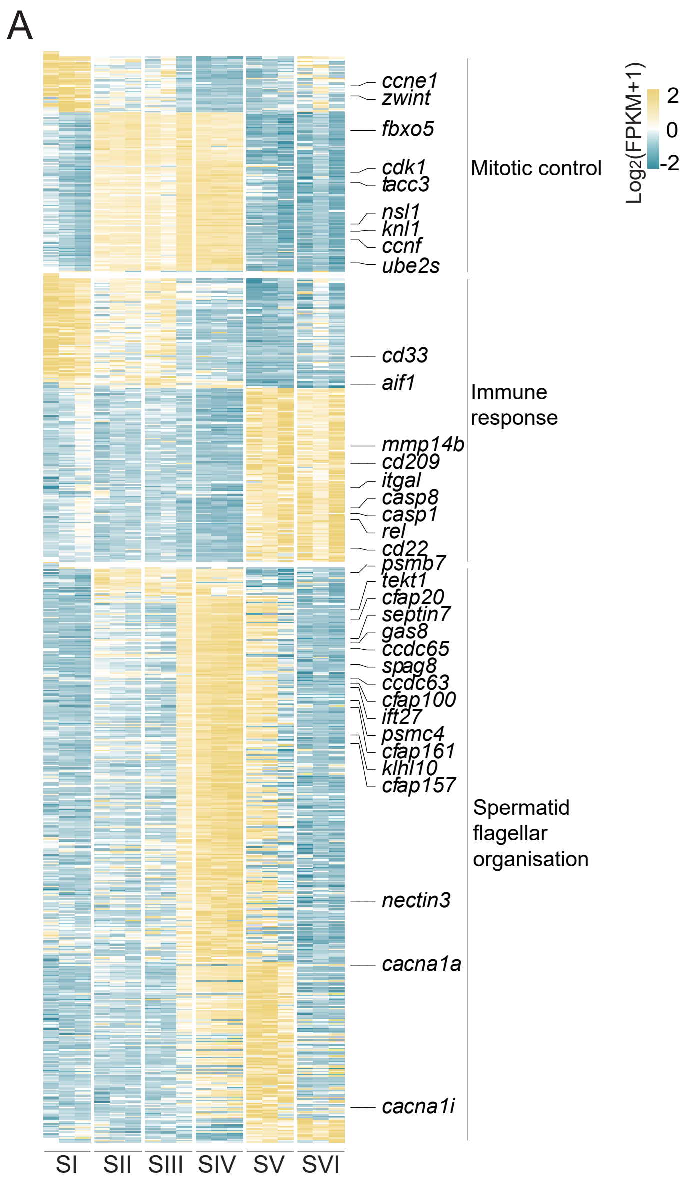

Supplementary Figure 7. DEGs grouped by mitotic control, immune response, and spermatid flagellar organisation. Heatmap of DEGs arranged by peak expression and functional category. Values represent row scaled Z scores of $\log_2$(FPKM + 1).
