# Supplementary material for: An integrated roadmap of European sea bass (Dicentrarchus labrax) spermatogenesis across the annual reproductive cycle
Source: Front Cell Dev Biol. 2026 Jun 24;14:1852477. doi: 10.3389/fcell.2026.1852477 (PMC13342237; doi:10.3389/fcell.2026.1852477)
Supplement: Supplementary file 15 [file Image1.pdf]

Supplementary Figure 1

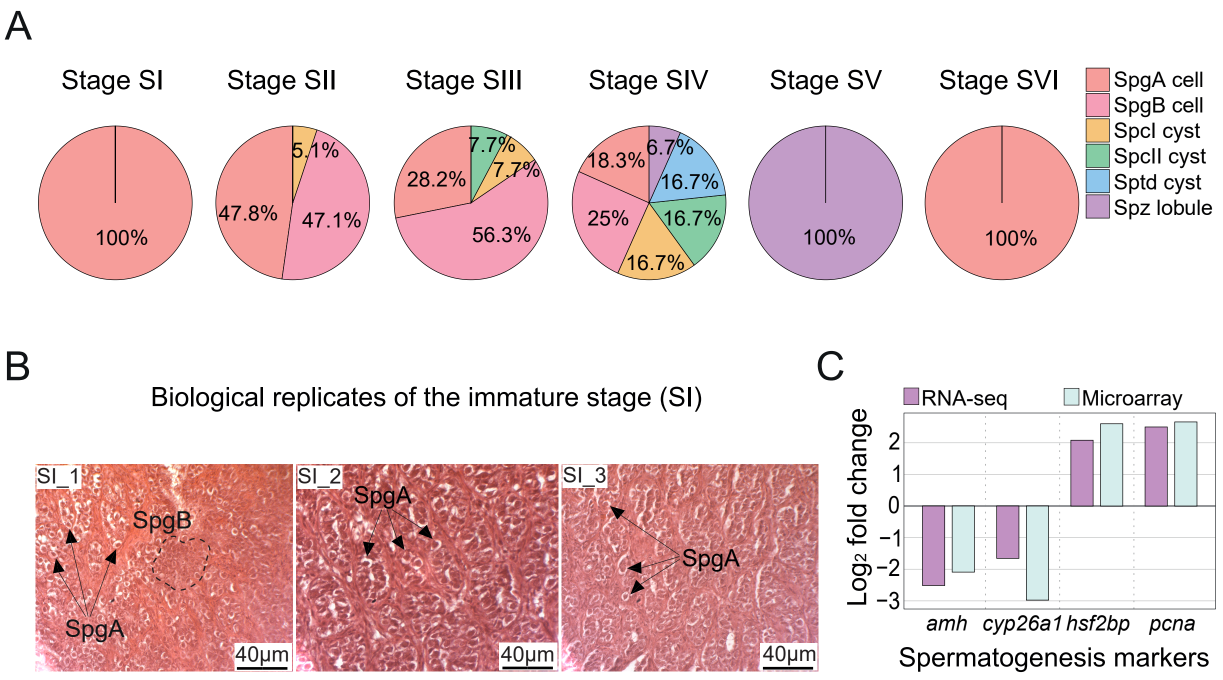

Supplementary Figure 1. Replicate structure and marker validation for the immature testis (SI). **(A)** Relative abundance of SpgA, SpgB, SpcI, SpcII, Sptd, and Spz across SI-SVI stages, quantified per lobule (H&E stain; ×40 magnification; scale bar, 40 µm). **(B)** Representative micrographs of SI biological replicates. SI_1 contains a small SpgB cyst. **(C)** Log2 fold-change comparison of early-stage markers (*amh, cyp26a1, pcna, hsf2bp*) between RNA-seq and microarray data (Blázquez et al. 2017).
